# Aspirin and Risk of Dementia in Patients with Late-Onset Depression: A Population-Based Cohort Study

**DOI:** 10.1155/2020/1704879

**Published:** 2020-01-29

**Authors:** Ya-Hsu Yang, Chih-Chiang Chiu, Hao-Wei Teng, Chun-Teng Huang, Chun-Yu Liu, Ling-Ju Huang

**Affiliations:** ^1^Department of Psychiatry, Taipei City Hospital, Renai Branch, Taipei, Taiwan; ^2^National Yang-Ming University, School of Medicine, Taipei, Taiwan; ^3^Department of Psychiatry, Taipei City Hospital, Songde Branch, Taipei, Taiwan; ^4^Department of Psychiatry, School of Medicine, Taipei Medical University, Taipei, Taiwan; ^5^Division of Medical Oncology, Department of Oncology, Taipei Veterans General Hospital, Taipei, Taiwan; ^6^Division of Hematology & Oncology, Department of Medicine, Yang-Ming Branch of Taipei City Hospital, Taipei, Taiwan; ^7^Division of General Medicine, Department of Medicine, Taipei Veterans General Hospital, Taipei, Taiwan

## Abstract

**Background:**

Late onset depression (LOD) often occurs in the context of vascular disease and may be associated with risk of dementia. Aspirin is widely used to reduce the risk of cardiovascular disease and stroke. However, its role in patients with LOD and risk of dementia remains inconclusive. *Materials and Methods.* A population-based study was conducted using data from National Health Insurance of Taiwan during 1996–2009. Patients fulfil diagnostic criteria for LOD with or without subsequent dementia (incident dementia) and among whom users of aspirin (75 mg daily for at least 6 months) were identified. The time-dependent Cox proportional hazards model was applied for multivariate analyses. Propensity scores with the one-to-one nearest-neighbor matching model were used to select matching patients. Cumulative incidence of incident dementia after diagnosis of LOD was calculated by Kaplan–Meier Method.

**Results:**

A total of 6028 (13.4%) and 40,411 (86.6%) patients were defined as, with and without diagnosis of LOD, among whom 2,424 (41.9%) were aspirin users. Patients with LOD had more comorbidities such as cardiovascular diseases, diabetes, and hypertension comparing to those without LOD. Among patients with LOD, aspirin users had lower incidence of subsequent incident dementia than non-users (Hazard Ratio = 0.734, 95% CI 0.641–0.841, *p* < 0.001). After matching aspirin users with non-users by propensity scores-matching method, the cumulative incidence of incident dementia was significantly lower in aspirin users of LOD patients (*p* < 0.001). After matching aspirin users with non-users by propensity scores-matching method, the cumulative incidence of incident dementia was significantly lower in aspirin users of LOD patients (

**Conclusions:**

Aspirin may be associated with a lower risk of incident dementia in patients with LOD. This beneficial effect of aspirin in LOD patients needs validation in prospective clinical trials and our results should be interpreted with caution.

## 1. Introduction

Late-onset depression (LOD) and cognitive impairment have emerged as important public health issues among the elderly following a global trend in population aging [1, 2]. LOD is a subtype of depression that commonly occurs in later life, with age of onset ranging from 50 to 65 year-old [3, 4]. Studies have shown LOD is closely associated with subsequent dementia [5, 6]. It has been reported that about half of the patients with LOD have cognitive impairment, whereas depression has been proposed to be both a risk factor and a prodrome for incident dementia [6, 7]. In addition, case-control studies have revealed a positive correlation between depression and subsequent onset of Alzheimer's disease in patients with LOD [8]. A recent meta-analysis revealed that baseline depression severity, co-morbid anxiety, executive dysfunction, current episode duration, early improvement, physical illnesses and age were statistically significant predictors of treatment outcomes of patients with LOD [9].

Various factors such as genetic factors, vascular changes, pre-existing medical, and neurological disorders may contribute to depression in the elderly, possibly from complex interactions of these factors [10]. Interestingly, LOD frequently arises in the context of vascular disorders [10–12], and it has been proposed complex vulnerability models involving endothelial dysfunction interacting with other complex multidirectional ways to mediate depression [13, 14]. Previously, we have used the NHIRD database to study the association of statin with risk of dementia [15]. We found that statins may reduce the risk of subsequent dementia in patients with LOD [15]. Similarly, Wu et al. have also reported the association of statins with reduced risk of dementia in elderly patients [16]. Although the definite effects of statins on risk of dementia remain to be elucidated, supporting evidence suggests statins may improve cognitive impairment in some patients, and may decrease the risk of dementia, Alzheimer's disease in some cases [17].

Aspirin (acetylsalicylic acid), a commonly used anti-inflammatory and antiplatelet agent, can interrupt neurotoxic cascades via its effects on inflammatory cascades, anti-platelet mechanisms [18], inhibiting cyclooxygenase (COX), and by suppressing production of prostaglandins and thromboxanes [19]. It is widely used to reduce the risk of atherosclerosis, heart disease, stroke, and potentially, some cancers [20–22]. However, its role in prevention of incident dementia in the elderly remains inconclusive. Some studies have reported that aspirin can prevent dementia, but others have not [18]. Few studies have explored the effects of aspirin in preventing subsequent incident dementia in patients with LOD.

We conducted a nationwide population-based study aiming to assess the use of aspirin in association with the risk of dementia in patients with LOD.

## 2. Methods

### 2.1. Data Sources

This study was approved by the Institutional Review Board of Taipei Veterans General Hospital, Taiwan. We used the Longitudinal Health Insurance Database (LHID) 2005 collated from the National Health Insurance Research Database (NHIRD), which was released by the National Health Research Institute in Taiwan. The National Health Insurance program finances health care for 99% of all of residents of Taiwan (>25 million enrollees). LHID 2005 contains all original claims data of 1,000,000 beneficiaries included in year 2005. They selected data, by random sampling, from the 2005 Registry for Beneficiaries (ID) of the NHIRD, in which registration data of every beneficiary of the National Health Insurance program during the period of January 1, 2005 to January 1, 2006 were recorded. The LHID 2005 included comprehensive information about insured people, including demographic data, dates of clinical visits, diagnostic codes, details of prescriptions expenditure levels and dates of enrolment and withdrawal between January 1996 and December 2009. There was no significant difference in the gender distribution (*Χ*^2^ = 0.008, *df* = 1, *p*-value = 0.931) between the enrollees listed in the LHID 2005 and those originally included under NHIRD (http://nhird.nhri.org.tw/en). Codes from International Classification of Diseases, 9th revision, Clinical Modification (ICD-9-CM) were used in LHID 2005.

Previously, we have used the NHIRD database to study the association of statins with risk of dementia [15]. Current study represents a new analysis from the NHIRD. We aimed to dissect the impact of different factors on the risk of dementia in patients with late onset depression (LOD). In our previous paper [15], the study object is anti-lipid agents (statins), whereas our current target of interest is aspirin. We used the same criteria to sort out the vulnerable population (patients with LOD, and with or without subsequent dementia), but we searched a population with different risk factor (who had taken aspirin). The aspirin users were not necessarily overlapped with statin users reported in previous literature [15].

The dataset used in this study consists of de-identified secondary data released to the public for research purposes. Personal information, including family history of cancer, lifestyle factors, and habits such as smoking and alcohol use, were not available from the NHIRD.

### 2.2. Study Sample

We conducted a retrospective cohort study from January 1, 1996 to December 31, 2009 and finally 46,439 individuals aged ≥65 years were included.

### 2.3. Identification of LOD, Dementia and Aspirin-Taking History

Subjects aged ≥65 years who had filed at least three service claims between 1996 and 2009 for either outpatient or inpatient care with a principal diagnosis of depression (ICD-9-CM: 311, 296.2, 296.3, and 300.4), and received concurrent treatment with anti-depressants, were identified as patients with LOD [15]. Similarly, those with a principal diagnosis of dementia (ICD-9-CM 331 290.0–290.4 and 294.1) were identified as patients with dementia. We excluded patients who had depression before 65 years of age and those without treatment with anti-depressants. Participants with a diagnosis of dementia at time of diagnosis of LOD (i.e., prevalent dementia) were excluded and only those who developed dementia after the diagnosis of LOD (i.e., incident dementia) were included at the beginning of data sorting. “Aspirin use” was defined as the use of aspirin during the entire study period in patients without dementia, or before the diagnosis of dementia, with a dosage of ≥75 mg per day for duration of ≥6 months.

### 2.4. Demographic Variables and Comorbidities

The demographic variables used in this study included age, gender, diabetes mellitus (DM) (ICD9-CM: 250), hypertension (HTN) (ICD9-CM: 401–405), chronic obstructive pulmonary disease (COPD) (ICD9-CM: 490–496), chronic renal insufficiency (CRI) (ICD9-CM 585), ischemic heart disease (IHD) (ICD9-CM:410–414), congestive heart failure (CHF) (ICD9-CM:428–429), and cerebrovascular accident (CVA) (ICD9-CM:430–436). Those study subjects who had filed at least three service claims between 1996 and 2009 for either outpatient or inpatient care with DM, HTN, COPD, CRI, IHD, CHF, and CVA were identified as patients with comorbidities.

### 2.5. Statistical Analysis

All included patients were followed until the end of 2009 unless one of the following occurred: diagnosis of dementia, death, or dropout from the National Health Insurance program. We estimated the risk of dementia among the study cohort using age- and gender-adjusted standardized incidence ratios (SIRs). The 95% CIs for the SIRs were estimated under the assumption that the observed number of dementia cases followed a Poisson probability distribution. The *t*-test was used to compare the means of two independent continuous samples. Categorical variables were compared using the chi-square (*χ*^ 2^) test between patients. The time dependent Cox proportional hazards model was applied for multivariate analyses to determine the adjusted hazard ratios (aHR) for dementia with aspirin use in patients with LOD. Propensity score methods were used to control for selection bias, [18] and derived using binary logistic regression to generate a propensity score for each patient who takes aspirin or not. The demographic variables entered in the propensity model were as previously mentioned. Subsequently, a one-to-one match between patients who received aspirin and those who did not receive aspirin was obtained using the nearest-neighbor matching method [18]. The paired *t*-test was used for comparing differences in continuous outcomes between treated and untreated subjects in the matched sample, while McNemar's test can be used to compare proportions. Comparable methods exist for relative risks. For time-to-event outcomes, Cox proportional hazards models stratified on the matched pairs, can be employed. Kaplan–Meier estimate with Log rank test was used to measure the fraction of patients with dementia living for a specified time after diagnosis of LOD. All statistical analyses were performed with SAS (version 9.3; SAS Institute, Cary, NC) and SPSS (version 18; SPSS institute, Chicago, IL). A *p*-value below 0.05 was considered statistically significant.

## 3. Results

### 3.1. Baseline Characteristics and Incidence of Subsequent Dementia between Included Cases with or without LOD

A total of 6028 (13.4%) and 40,411 (86.6%) patients were defined as with and without diagnosis of LOD. Similar to our prior report, Patients with LOD were significantly older, predominantly female, and had a higher prevalence of COPD, DM, HTN, IHD, CHF, CVA, and CRI (as shown in Supplementary [Supplementary-material supplementary-material-1]). Further, patients with LOD were more likely to develop dementia during the follow-up period compared with those without LOD (18.8% vs. 13.6%, *p* < 0.001). The age- and gender-adjusted SIRs of dementia for patients with LOD was 2.786 (95% CI, 2.627–2.953). The mean follow-up duration for patients without LOD was 13.27 ± 2.53 years, and 5.98 ± 3.50 years for patients with LOD (Supplementary [Supplementary-material supplementary-material-1]).

### 3.2. Baseline Characteristics and Incidence of Subsequent Dementia between Patients of LOD with or without Aspirin Use

We further divided the patients with LOD according to aspirin use.  Table 1 shows characteristics of aspirin users and non users among patients with LOD. The frequency of aspirin use in patients with LOD is 58.1% (3604 patients). Compared to aspirin nonusers, aspirin users were significantly older, more male, and had a higher prevalence of COPD, DM, HTN, IHD, CHF, CVA, and CRI, suggesting indication use of aspirin for patients with comorbidities under reimbursement regulation. Moreover, the mean time to dementia diagnosis in patients with aspirin is 3.26 years versus that in those without aspirin is 2.94 years (*p* < 0.001), respectively. Next we examined difference of comorbidities in the patients with LOD according to diagnosis with or without subsequent incident dementia (Table 2). Table 2 shows that patients with LOD who developed subsequent dementia had a higher prevalence of COPD, DM, HTN, IHD, CHF, CVA, and CRI.

### 3.3. Relative Risk of Dementia in LOD Patients

Multivariate time-dependent Cox proportional-hazards regression analysis was carried out to estimate the aHR of subsequent dementia in LOD patients (Table 3). After controlling potential confounders including all demographic variables, aspirin users had a significantly lower risk of dementia than aspirin nonusers (HR = 0.734, 95% CI 0.641–0.841, *p* < 0.001). In addition, age, female gender and comorbidities such as DM, HTN, COPD, and CVA were significant risk factors of dementia in patients with LOD (Table 3).

### 3.4. Factors Associated with Dementia after Propensity Score Correction Using the One-To-One Nearest-Neighbor Matching Method

**Figure 1:**
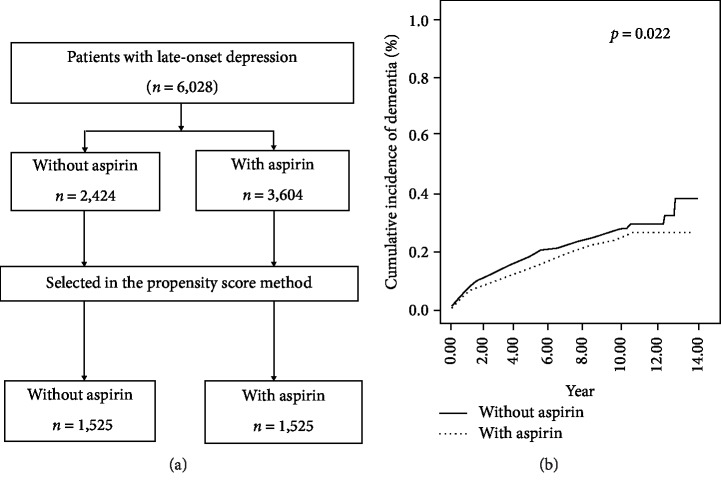
Comparison of the risk of subsequent dementia in patients with late-onset depression, based on aspirin use. (a) Flowchart showing the matching process for aspirin users and non-users in the studied. Finally, 1,525 pairs of matched patients were selected for analysis using the propensity score method, which minimizes interference by confounding factors. (b) Aspirin reduced the occurrence of subsequent dementia in patients with LOD (*p* = 0.022).

To validate the potential benefits of aspirin in patients with LOD for preventing dementia, we applied the propensity score correction method using one-to-one nearest-neighbor matching to minimize the confounding factors, including all demographic variables (Figure 1(a)). Aspirin users may also have other known risk factors for dementia, such as DM. Hence, we need to establish if aspirin per se prevents subsequent dementia. A total of 1,525 patients from each group were matched for the aforementioned factors, which appeared well-matched between groups (Table 4). On subsequent analysis, we found that aspirin decreased the risk of dementia significantly (Figure 1(b)) (*p* = 0.022), and was an independent protective factor against dementia by using multivariate analysis (Table 5). The aHR for aspirin users was 0.833 (95% CI 0.708–0.981, *p* = 0.029). Only aspirin use, age, DM, HTN, COPD, and CVA remained independent risk factors for dementia after adjustment in this analysis.

## 4. Discussion

This study demonstrates that aspirin use is associated with a lower risk of incident dementia. In addition, our study also supports the previous finding that LOD is a risk factor for subsequent dementia [15].

In the EURODEM study, depression has been shown to be a risk for subsequent Alzheimer's disease onset, with an OR of 1.82 with a 95% CI of 1.16–2.83 [8, 23]. This correlation was confined to patients with LOD (OR 2.44; 95% CI 1.36–4.36). This finding has been further supported by a large-scale cohort study and meta-analysis [24]. However, the study did not find an association between antidepressant treatment and Alzheimer's disease in patients with depression [24]. In our current study, the prevalence of LOD among elderly individuals in Taiwan was 13.4%, which is similar as previous reports [25]. One explanation for this high prevalence is that the rates are at least doubled among patients with handicap or disability and those in hospital or nursing homes. Depressive symptoms are not uncommon in elderly patients with chronic comorbidities [26]. The other explanation is that Taiwan's universal National Health Insurance covers more than 98% of Taiwan's population and enrollees enjoy almost free access to healthcare with small co-payment by most clinics and hospitals. In addition, the age and gender-adjusted SIRs of dementia in patients with LOD was 2.786 (95% CI 2.627–2.953). In a previous retrospective cohort study, Buntinx et al. found the HR of LOD patients with subsequent dementia was 2.55 when compared with those without LOD (95% CI 1.19–5.47) [27]. Our study also pointed out that LOD was a risk factor for dementia in older patients [15].

Nilsson et al. found that aspirin users had a significantly lower prevalence of Alzheimer's dementia and maintained better cognitive function than non-users, in both the cross-sectional and longitudinal analyses [28]. Some studies had also reported that aspirin may preserve cognitive function and confer protection against Alzheimer's dementia [29, 30], contrast to some other studies did not show protective effects of aspirin on cognitive functions [31–33]. Therefore the benefit of aspirin in dementia patients has remained inconclusive due to conflicting studies. Our study discovered the use of aspirin in LOD patients is associated with reduced risk of the subsequent onset of dementia. In addition, we found no duration-dependent effect of aspirin on dementia incidence in our study. The protective effect of aspirin seemed to be maintained in patients taking aspirin for 2 years or longer (the HR was 0.810, *p* = 0.002). The possible reason is that we could not define the severity of comorbidity and those patients who were taking “long-duration” aspirin might also have more severe comorbidities, since aspirin has been indicated for patients with cardiovascular disease, or patients who are at risk of developing cardiovascular or stroke events (such as diabetes, hyperlipidemia, etc.) under the reimbursement by Taiwan NHI.

Previous study reported that depression can be divided into three groups: (1) early-onset with longstanding psychobiological vulnerability; (2) late-onset as reaction to severe life stress; and (3) late-onset with vascular risk factors [12]. From a growing body of evidence, it is well-established that LOD differs from early onset dementia. Patients with LOD are more likely to have no family history of mood disorders, fewer premorbid personality disorders, more vascular risk factors with concomitant cognitive impairment, and no response to the initial use of anti-depressants [12, 34, 35]. As stated earlier, a substantial proportion of patients with LOD are associated with vascular risk factors [10–12]. Moreover, the potential protective effects of aspirin on vascular dementia have been reviewed by literature [36, 37]. In this study, older people with LOD (Age ≥65 years) were enrolled and a higher prevalence of cardiovascular disease was noted. This may partially explain why aspirin users had a reduced risk for subsequent dementia in LOD patients. In addition, some studies discovered inflammatory processes and oxidative stress may mediate both depression and vascular disease via neurotoxic cascades [38].Aspirin, a non-steroidal anti-inflammatory drug, can reduce oxidative stress and protect against oxidative damage by stimulating endogenous production of anti-inflammatory regulatory “braking signals”, which reduce inflammatory responses and levels of inflammatory biomarkers [36, 37].

Despite current study demonstrated aspirin associated with a lower risk of incident dementia in patients with LOD, previously the Aspirin in Reducing Events in the Elderly (ASPREE) trial in contrast reported a higher all-cause mortality among healthy older adults who received daily aspirin than among those who received placebo and this increased mortality could be attributed primarily to cancer-related death [39]. ASPREE, conducted in Australia and the United States with a total of 19,114 relatively healthy older participants from community settings, is a primary prevention trial investigating the potential benefits of daily use of 100 mg of enteric-coated aspirin [39–41]. The trial endpoints included disability-free survival, defined as survival free from dementia or persistent physical disability. The ASPREE trial did not support a universal primary prevention strategy of low-dose aspirin in a healthy elderly population [39–41]. However, the ASPREE results did not conflict with the established USPSTF guidelines supporting the secondary preventive use of aspirin among people with a prior history of a vascular event or primary prevention for individuals aging 50–59 years with a 10-year risk of a cardiovascular event greater than 10% [42, 43]. Indeed, in Taiwan National Health System, the prescription of aspirin is largely in compliance with indication usage, which includes thrombosis prevention in patients with significant risks for cardiovascular or cerebrovascular events and secondary prevention in patients with established ischemic heart disease. The thrombosis-preventing advantage of aspirin is encountered by the increased hemorrhage risk of this drug and therefore any primary preventive approach using low dose aspirin to lower disease risk should be carefully examined by well-designed prospective trials [44]. Collectively, the potential beneficial effects of aspirin in lowering incident dementia in patients with LOD needs to be validated by prospective controlled clinical trials in future.

## 5. Strengths and Limitations

The strengths of our study include a large number of patients, a long follow-up period, and detailed records of co-morbid diseases. Our study discovered that the use of aspirin in LOD patients is associated with reduced incident dementia, implicating a potential benefit of aspirin in slowing cognitive decline in patients with LOD. Based on current study, the effect of aspirin is limited to patients with LOD and whether it applies more broadly remains unclear and more studies are needed. This study also has the following limitations: (1) several factors affecting LOD and dementia, such as socioeconomic status, drug-taking compliance, lifestyle, obesity, smoking, alcohol use, the use of vitamin or fish oil supplementation, and family history could not be assessed from this dataset, and some residual confounders may be exerting their effects; (2) the subtypes of dementia were not revealed by the dataset, so we could not determine whether the protective effects of aspirin are the same in different subtypes of dementia; (3) information on some important depression-related variables, such as severity of comorbid disease, severity of depression, frequency of depressive episodes, comorbid psychotic features, was not available; (4) given the nature of this research database, it is difficult for us to estimate the number of dropouts of this study; (5) detection of dementia is likely to be influenced by the number of clinical contacts; unfortunately, we were not able to retrieve such information from the database we used; (6) despite common comorbidities such as congestive heart failure, chronic obstructive pulmonary disease, chronic renal insufficiency, cerebrovascular accident, diabetes mellitus, hypertension, ischemic heart disease have been controlled factors for current analysis, other comorbidities may also have potential impact on our analysis; (7) last but not the least, we could not follow patients' psychiatric therapeutic courses, it is also possible that other than aspirin, variations in medication for LOD might have generated an unexpected bias in current analysis that has tilted the conclusion. However, given the relatively large number of cases we speculate that the variation of medication for depression may not differ significantly between aspirin and non-aspirin groups after propensity score matching.

## 6. Conclusion

Aspirin may be associated with a lower risk of incident dementia in patients with LOD. However, in the context of ASPREE trial demonstrating the negative effects of preventive low dose aspirin use in healthy elderly population, our finding should be interpreted with caution. Double-blind randomized controlled trials are warranted to validate this finding before putting it into clinical practice.

## Figures and Tables

**Table 1:** Characteristics of aspirin users and non-users among patients with LOD (*n* = 6.028).

		Aspirin nonuser		Aspirin user		*p*-value
		*n* = 2, 424 (41.9%)		*n* = 3, 604 (58.1%)		
Age (years)	Mean (SD)	72.47	(5.74)	73.58	(5.67)	<0.001
Gender	F	1,451	(59.9)	2,007	(55.7)	0.001
	M	973	(40.1)	1,597	(44.3)	
COPD	No	1,210	(49.9)	1,453	(40.3)	<0.001
	Yes	1,214	(50.1)	2,151	(59.7)	
DM	No	1,734	(71.5)	1,985	(55.1)	<0.001
	Yes	690	(28.5)	1,619	(44.9)	
HTN	No	708	(29.2)	255	(7.1)	<0.001
	Yes	1,716	(70.8)	3,349	(92.9)	
IHD	No	1,693	(69.8)	1,125	(31.2)	<0.001
	Yes	731	(30.2)	2,479	(68.8)	
CHF	No	2,009	(82.9)	2,331	(64.7)	<0.001
	Yes	415	(17.1)	1,273	(35.3)	
CVA	No	1,842	(76.0)	1,635	(45.4)	<0.001
	Yes	582	(24.0)	1,969	(54.6)	
CRI	No	2,151	(88.7)	3,038	(84.3)	<0.001
	Yes	273	(11.3)	566	(15.7)	
Follow-up duration (year)	Mean ± SD	5.8 ± 3.52		6.18 ± 3.46		<0.001

CHF, congestive heart failure; COPD, chronic obstructive pulmonary disease; CRI, chronic renal insufficiency; CVA, cerebrovascular accident; DM, diabetes mellitus; HTN, hypertension; IHD, ischemic heart disease; LOD, late-onset depression; SD, standard deviation. The *t*-test was used to comparing the means of age and duration. Categorical variables (gender, COPD, DM, HTN, IHD, CHF, CVA, CRI, and dementia) were compared using the Chi-square test between patients.

**Table 2:** Characteristics of LOD patients with or without subsequent dementia (*n* = 6, 028).

		Without dementia		With dementia		*p*-value
		*n* = 4, 892 (81.2%)		*n* = 1, 136 (18.8%)		
Age (years)	Mean (SD)	72.47	(5.64)	74.58	(5.81)	<0.001
Gender	F	2,785	(56.9)	673	(59.2)	0.156
	M	2,107	(43.1)	463	(40.8)	
COPD	No	2,251	(46.0)	412	(36.3)	<0.001
	Yes	2,641	(54.0)	724	(63.7)	
DM	No	3,056	(62.5)	663	(58.4)	0.010
	Yes	1,836	(37.5)	473	(41.6)	
HTN	No	835	(17.1)	128	(11.3)	<0.001
	Yes	4,057	(82.9)	1,008	(88.7)	
IHD	No	2,328	(47.6)	490	(43.1)	0.007
	Yes	2,564	(52.4)	646	(56.9)	
CHF	No	3,555	(72.7)	785	(69.1)	0.016
	Yes	1,337	(27.3)	351	(30.9)	
CVA	No	2,982	(61.0)	495	(43.6)	<0.001
	Yes	1,910	(39.0)	641	(56.4)	
CRI	No	4,238	(86.6)	951	(83.7)	0.011
	Yes	654	(13.4)	185	(16.3)	

CHF, congestive heart failure; COPD, chronic obstructive pulmonary disease; CRI, chronic renal insufficiency; CVA, cerebrovascular accident; DM, diabetes mellitus; HTN, hypertension; IHD, ischemic heart disease; LOD, late-onset depression; SD, standard deviation. The *t*-test was used to comparing the means of age. Categorical variables (gender, COPD, DM, HTN, IHD, CHF, CVA, CRI) were compared using the Chi-square test between patients.

**Table 3:** Risk factors for subsequent dementia in LOD patients as determined using a multivariate time to dependent Cox proportional hazards model (*n* = 6, 028).

	Hazard ratios	95% CI	*p*-value
Aspirin			
Yes *vs.* No	0.734	(0.641–0.841)	<0.001
Age			
Elder *vs*. younger	1.064	(1.054–1.074)	<0.001
Gender			
Female *vs*. male	1.211	(1.073–1.366)	0.002
DM			
Yes *vs*. No	1.142	(1.012–1.289)	0.032
HTN			
Yes *vs.* No	1.237	(1.018–1.503)	0.032
COPD			
Yes *vs.* No	1.268	(1.119–1.437)	<0.001
CRI			
Yes *vs.* No	1.063	(0.906–1.247)	0.453
IHD			
Yes* vs.* No	1.064	(0.935–1.211)	0.348
CHF			
Yes *vs.* No	0.939	(0.821–1.072)	0.351
CVA			
Yes *vs.* No	1.684	(1.487–1.907)	<0.001

CHF, congestive heart failure; COPD, chronic obstructive pulmonary disease; CRI, chronic renal insufficiency; CVA, cerebrovascular accident; DM, diabetes mellitus; HTN, hypertension; IHD, ischemic heart disease; LOD, late-onset depression.

**Table 4:** Comparison of the baseline demographics in patients with LOD after matching by propensity score method (*n* = 3, 050).

		Aspirin nonuser		Aspirin user		*p*-value
		*n* = 1, 525 (%)		*n* = 1, 525 (%)		
Age (years)	Mean (SD)	73.21	(5.87)	73.19	(5.52)	0.946
Sex	F	913	(59.9)	922	(60.5)	0.728
	M	612	(40.1)	603	(39.5)	
COPD	No	682	(44.7)	672	(44.14)	0.731
	Yes	843	(55.3)	853	(55.9)	
DM	No	956	(62.7)	963	(63.1)	0.777
	Yes	569	(37.3)	562	(36.9)	
HTN	No	208	(13.6)	210	(13.8)	0.946
	Yes	1317	(86.4)	1315	(86.2)	
IHD	No	819	(53.7)	815	(53.4)	0.880
	Yes	706	(46.3)	710	(46.6)	
CHF	No	1155	(75.7)	1140	(74.8)	0.442
	Yes	370	(24.3)	385	(25.2)	
CVA	No	978	(64.1)	983	(64.5)	0.846
	Yes	547	(35.9)	542	(35.5)	
CRI	No	1329	(87.1)	1331	(87.3)	0.935
	Yes	196	(12.9)	194	(12.7)	

CHF, congestive heart failure; COPD, chronic obstructive pulmonary disease; CRI, chronic renal insufficiency; CVA, cerebrovascular accident; DM, diabetes mellitus; HTN, hypertension; IHD, ischemic heart disease; LOD, late-onset depression; SD, standard deviation. The paired *t*-test was used for comparing the means of age, while McNemar's test can be used to compare proportions of gender, COPD, DM, HTN, IHD, CHF, CVA, and CRI.

**Table 5:** Hazard ratios of aspirin use for risk of subsequent dementia in patients with LOD multivariate Cox proportional hazards model after matching by propensity score (*n* = 3, 050).

	Hazard ratios	95% CI	*p*-value
Aspirin			
Yes *vs.* No	0.833	(0.708–0.981)	0.029
Age			
Elder *vs*. younger	1.056	(1.042–1.0701)	<0.001
Sex			
Female *vs*. male	1.063	(0.896–1.262)	0.485
DM			
Yes *vs*. No	1.266	(1.072–1.495)	0.006
HTN			
Yes *vs. *No	1.385	(1.032–1.787)	0.029
COPD			
Yes *vs. *No	1.222	(1.028–1.452)	0.023
CRI			
Yes *vs.* No	1.081	(0.862–1.357)	0.500
IHD			
Yes* vs.* No	1.106	(0.934–1.309)	0.241
CHF			
Yes *vs.* No	0.966	(0.825–1.204)	0.970
CVA			
Yes *vs.* No	1.945	(1.649–2.294)	<0.001

CHF, congestive heart failure; COPD, chronic obstructive pulmonary disease; CRI, chronic renal insufficiency; CVA, cerebrovascular accident; DM, diabetes mellitus; HTN, hypertension; IHD, ischemic heart disease; LOD, late-onset depression.

## Data Availability

The database may be accessed at: http://nhird.nhri.org.tw/en/index.htm.

## Supplementary Materials

Supplementary Materials Supplementary Table 1: Characteristics of patients with or without LOD.
