# Supplementary material for: Aspirin and Risk of Dementia in Patients with Late-Onset Depression: A Population-Based Cohort Study
Source: Biomed Res Int. 2020 Jan 29;2020:1704879. doi: 10.1155/2020/1704879 (PMC7008294; doi:10.1155/2020/1704879)
Supplement: Supplementary Materials — Supplementary Table 1: Characteristics of patients with or without LOD. [file 1704879.f1.docx]

| **Supplementary Table 1** Characteristics of patients with or without LOD | | | | | | |
| --- | --- | --- | --- | --- | --- | --- |
|  |  | Without LOD  *n* = 40,411 (86.6%) | | With LOD  *n* = 6,028 (13.4%) | | *p*-value |
| Age (years) | Mean(SD) | 71.08 | (4.85) | 73.41 | (5.73) | <0.001 |
| Gender | F | 18,845 | (46.6) | 3,458 | (57.4) | <0.001 |
|  | M | 21,566 | (53.4) | 2,570 | (42.6) |  |
| COPD | No | 21,198 | (52.5) | 2,663 | (44.2) | <0.001 |
|  | Yes | 19,213 | (47.5) | 3,365 | (55.8) |  |
| DM | No | 28,559 | (70.7) | 3,719 | (61.7) | <0.001 |
|  | Yes | 11,852 | (29.3) | 2,309 | (38.3) |  |
| HTN | No | 10,079 | (24.9) | 963 | (16.0) | <0.001 |
|  | Yes | 30,332 | (75.1) | 5,065 | (84.0) |  |
| IHD | No | 24,389 | (60.4) | 2,818 | (46.7) | <0.001 |
|  | Yes | 16,022 | (39.6) | 3,210 | (53.3) |  |
| CHF | No | 30,680 | (75.9) | 4,340 | (72.0) | <0.001 |
|  | Yes | 9,731 | (24.1) | 1,688 | (28.0) |  |
| CVA | No | 28,399 | (70.3) | 3,477 | (57.7) | <0.001 |
|  | Yes | 12,012 | (29.7) | 2,551 | (42.3) |  |
| CRI | No | 36,111 | (89.4) | 5,189 | (86.1) | <0.001 |
|  | Yes | 4,300 | (10.6) | 839 | (13.9) |  |
| Dementia | No | 34,926 | (86.4) | 4,892 | (81.2) | <0.001 |
|  | Yes | 5,485 | (13.6) | 1,136 | (18.8) |  |
| Follow-up duration (year) | Mean ± SD | 13.27 ± 2.53 | | 5.98 ± 3.50 | | <0.001 |
| Abbreviation: CHF, congestive heart failure; COPD, chronic obstructive pulmonary disease; CRI, chronic renal insufficiency; CVA, cerebrovascular accident; DM, diabetes mellitus; HTN, hypertension; IHD, ischemic heart disease; LOD, late-onset depression; SD, standard deviation. The t-test was used to comparing the means of age and duration. Categorical variables (gender, COPD, DM, HTN, IHD, CHF, CVA, CRI and dementia) were compared using the Chi-Square test between patients. | | | | | | |
